# Methodological Reflections from Engaging Five Culturally and Linguistically Unique U.S. Muslim Populations

**DOI:** 10.3390/healthcare14070935

**Published:** 2026-04-03

**Authors:** Asma Mahd Ali, Ejura Yetunde Salihu, Salma Abdelwahab, Olayinka O. Shiyanbola, Eva Vivian, Betty Chewning

**Affiliations:** 1 Health Systems Management and Policy, School of Public Health, The University of Memphis, Memphis, TN 38152, USA; 2 Department of Family Medicine and Community Health, University of Wisconsin–Madison, Madison, WI 53705, USA; ejura.salihu@fammed.wisc.edu; 3 IQVIA, Cairo 12311, Egypt; 4 Department of Clinical Pharmacy, University of Michigan, Ann Arbor, MI 48109, USA; yinka@umich.edu; 5 Clinical Practice, Innovation, and Research Division, School of Pharmacy, University of Wisconsin–Madison, Madison, WI 53705, USAbetty.chewning@wisc.edu (B.C.)

**Keywords:** community-engaged research, Muslims, limited English proficiency, language interpretation, recruitment of diverse populations

## Abstract

**Background**: Engaging diverse populations, including Muslims, in research activities is important to support patient-centered research and improve health equity. **Objectives**: The research aimed to describe the community engagement steps that informed conducting research with five distinctively diverse U.S. Muslim communities. **Methods**: This work provides methodological reflections on engaging diverse Muslim communities in the U.S. Researchers built trust-based partnerships with community healthcare organizations and engaged with administrative leaders, advisory members, and people from five diverse communities. Strategies to support sampling, recruitment, multi-language interpretation methods, and how to engage communities and address their concerns are discussed. **Results**: A total of 22 participants were included in the original study. The research team successfully engaged five of the six planned communities, utilizing multiple interpretation methods and participating in community events to support recruitment and relationship-building. Direct-to-participant recruitment efforts were strengthened by personal connections with trusted community members. **Conclusions**: Flexibility and adaptability are integral in recruitment and data collection, as diverse communities may respond differently to methods successfully used elsewhere. Attention to gender-related cultural norms, the inclusion of language-concordant researchers, and respect for communities’ autonomy in deciding whether and how to participate collectively contributed to more effective and culturally grounded engagement with Muslim communities.

## 1. Introduction

Engaging diverse and underrepresented populations in U.S. research can be challenging, including efforts to involve Muslim communities. A previous community-based participatory research (CBPR) study noted challenges in sustaining partnerships with Muslim community organizations and highlighted the need to culturally adapt research methods [1]. Although the researchers engaged several major community organizations in Michigan, they observed that limited prior research experience and varying organizational capacities influenced the extent to which these partners could prioritize and sustain involvement in the project [1].

Muslims are the most racially diverse religious group in the U.S. [2]. Diversity reflects the range of cultural, linguistic, and immigration-related experiences that shape individuals’ identities. About 20% of U.S. Muslims come from South Asia (Pakistan, Afghanistan), while 14% come from the Middle East and North Africa (MENA) [3].

Muslims from the same region may speak distinct languages and embrace different practices and cultures. Afghanistan’s main language is Dari, while Pakistan’s is Urdu [4]. Unlike Afghanistan, Pakistan’s cultural traditions tend to be influenced by India [5]. The Rohingya (from Myanmar in Southeast Asia) language is primarily oral and lacks a standardized written format [6]. Muslim Arabs are originally from the MENA region. Cultural differences and similarities are present between Arabs [7]. Half of the U.S. immigrant population has limited English proficiency (LEP) [8,9].

Language barriers significantly impact healthcare access, with over half of immigrants with LEP encountering difficulties when seeking medical services [9]. LEP contributes to delays in seeking care and hinders the development of patient–provider relationships [10]. Language barriers also impair effective communication, influencing suboptimal care experiences, dissatisfaction, treatment non-adherence, and delayed disease management, ultimately resulting in poorer health outcomes [10].

Given the size, diversity, and unmet healthcare needs of the U.S. Muslim immigrants and refugees, it is important to better understand their diverse healthcare requirements and experiences navigating the healthcare system to make care more culturally responsive and equitable.

## 2. Objectives

The parent study conducted qualitative research to compare healthcare experiences and challenges of Muslim populations with diabetes, considering their diverse cultural, linguistic, and immigration status characteristics within a single project [11]. The overall objective of this study is to investigate the challenges and facilitators of community-engaged research when the sample is composed of people with diverse languages and cultures. Although prior studies have included participants from different countries of origin [12,13], none have integrated all of the key elements represented in our sample. To our knowledge, no single study has simultaneously included non-English-speaking participants [13], individuals from diverse cultural backgrounds [14], and the specific communities engaged in the present research.

This paper aims to provide a methodological reflection on our implementation of the parent qualitative study, offering a detailed account of the recruitment and engagement processes, as well as the interview procedures incorporated within that project. Specifically, we describe the community engagement steps that informed (1) the sampling strategy; (2) multi-language interview methods responsive to cultural preferences; (3) recruitment processes; and (4) establishing legitimacy within the diverse communities ahead of recruitment. Together, these reflections highlight lessons learned from conducting research simultaneously across distinctly different Muslim populations. The University of Wisconsin’s Institutional Review Board determined the study to be exempt.

## 3. Methods

### 3.1. Community Engaged-Research Design Process

Community-engaged research depends on partnerships with communities [15]. Guided by the 10-step patient engagement framework [16,17], early in the study planning process, the research team leveraged existing partnerships with two Muslim community organizations serving underrepresented populations and Muslims in Milwaukee: a community health and senior center, and a locally owned community pharmacy system composed of 21 pharmacies. Their advice informed several early key study decisions (e.g., the communities and languages to be included) and continued to guide the research through monthly meetings. During these meetings, researchers reported progress and sought advice to find strategic solutions to challenges encountered during the study. Additional community-engaged research methods were guided by the Sustaining Engagement of Blended Stakeholder Boards toolkit and included the establishment of two advisory groups—one consisting of clinicians and the other of community members. These groups provided guidance on various design decisions [16,17,18,19]. The clinician advisory group was composed of three racially diverse (Arab, Asian, White) pharmacists and a physician assistant (Arab). The community advisory group members included three racially diverse (African American, Arab, North African) people with diabetes and a case manager as a representative of the Rohingya community (i.e., a professional who assists newly arrived refugees in integrating into their new community by providing support with housing, healthcare, education, and employment). The team held a formal meeting with the group at the beginning of the project to understand the community’s needs and to design the qualitative study accordingly. As the project continued, the PI met informally with each member individually to inform them of the progress of the project. Finally, ongoing interactions with community leaders and advocates offered key entry points and strategies for gaining community trust. All advisory group members were compensated for their participation.

### 3.2. Sampling Strategy

During our initial meeting with our community health center partner, they reported that 31% of individuals receiving care at the clinic had a diagnosis of diabetes. Of those cases, 98% were classified as type 2 diabetes. They also identified the six largest groups in their health system to capture the diversity of the U.S. Muslim community. These details influenced the decision to intentionally select people from these groups according to their primary language spoken at home, reflecting the country of origin [11]. This approach ensured the inclusion of hardly reached, non-English-speaking people whose experiences are rarely captured in U.S. research [11]. Primary languages included Arabic (people from the Middle East), Dari (Afghan refugees), English (African American and White people), Rohingya (refugees from Myanmar), Somali (people from the African country Somalia), Urdu (people from Pakistan and India) [11]. Although there are cultural differences between these groups, the Islamic religion connects all of them as one Ummah (i.e., community). Basic Islamic ethical and interaction manners (e.g., justice, respect, compassion, humility, dignity, and integrity) were important in phrasing the research purpose, sampling, and communicating with this diverse group of people [20]. Eligibility criteria also included being an adult Muslim man or woman residing in the United States with a diagnosis of type 2 diabetes and at least six months of oral or injectable diabetes treatment [11].

We planned to recruit 30 people, five participants from each of the spoken languages; the sample size was based on information power [21]. More comprehensive details regarding the sample size are provided in a separate article [11].

### 3.3. Multi-Language Interview Methods

#### 3.3.1. Interview Procedures

A semi-structured interview guide with probes was developed to inquire about different domains of interest guided by diabetes self-management activities (e.g., access to medication, diabetes management-related behaviors, including dieting, exercising, checking blood sugar levels, and medication use) [22]. Questions were revised by experts in qualitative research and by the members of both advisory groups. The advisory group reviewed the questions to reflect their communities’ priorities [23] and the experiences of the heterogeneous communities. For instance, the refugee case manager highlighted access to care challenges faced by the Rohingya refugees. Interviews were planned for 60 minutes. Interviews were audio-recorded. All interviews except the ones with the Arabic speakers were transcribed verbatim by a professional service provider.

#### 3.3.2. Interpreter Modality

Three forms of interpretation took place throughout this study: phone interpretation from a vendor, in-person interpretation from the Rohingya community, and an in-person interview by a bilingual researcher. By-phone professional interpretation was provided by a vendor contractor with the University of Wisconsin–Madison for all the Dari- and Urdu-speaking participants and for two of the Rohingya participants. Three out of the five Rohingya interviews were facilitated by an in-person interpreter who is a community member and assisted in participant recruitment. When an interpreter was present, A.A. asked each question in English, the interpreter conveyed it to the participant in their language, and after the participant responded, the interpreter translated the response back into English [11]. A.A. is bilingual and conducted all interviews with Arabic-speaking participants in Arabic. A.A. translated the Arabic interviews during the transcription process. While A.A. translated the Arabic interviews, S.A. verified the translations by reviewing the recordings and corresponding transcripts to ensure accuracy. More details on transcribing and translation are available in a separate article [11].

To ensure that interpreters were not paraphrasing the information provided by the participants, the interviewer briefed interpreters about research objectives, expectations, and the importance of interpreting what participants say without paraphrasing. Although the interviewer was not familiar with the languages, she was alert for cues of misinterpretation and would ask the interpreter for clarification if it appeared that paraphrasing had occurred.

#### 3.3.3. Researcher Positionality

A.A. is a Middle Eastern Muslim woman, a second-generation Palestinian refugee, and a first-generation U.S. immigrant. On a regular basis, A.A. wore a Hijab as a Muslim, and she was aware that a respectful practice of attending a mosque is to wear an Abaya (long, modest dress with full sleeves).

## 4. Results

A total of 22 eligible participants were interviewed [11]. A full description of participant demographic characteristics is presented in another article [11]. Interviews took place between 27 July 2022 and 18 January 2023. Although interviews were initially planned for 60 minutes, some interviews took up to 90 min because of technical difficulties, described later in this manuscript.

### 4.1. Recruitment Processes

Building on partnering organizations’ advice and referrals, the goal was to recruit individuals from hardly reached populations while being culturally responsive and sensitive. A.A. had a pivotal role in this recruitment process. The most successful recruitment strategies stemmed from trust-based relationships; drawing on this approach, the team was able to recruit and interview participants from 5 of the 6 target communities. Twenty-two interviews were conducted. To accomplish this, the research team pursued the following strategies to build trust and recruit eligible participants.

#### 4.1.1. Finding Where People from Each Community Are Located and Understanding Their Characteristics

Engagement with community advisory groups and representatives from healthcare partnering organizations enabled the team to identify each community’s specific needs, available resources, and cultural practices. This information guided decisions about optimal recruitment locations and outreach approaches. For instance, some communities were mainly refugees and had established roots, having been in the U.S. for a longer period than others. The Rohingya community had an established mosque that specifically caters to the Rohingya people in their own language. On the other hand, the Afghan community does not have an established mosque as the majority have been recently resettled in Wisconsin. For this community, the team identified organizations that focus on supporting their unique needs, such as creating a sense of community and providing English learning programs for the youth and their mothers.

#### 4.1.2. Approaching Communities and Establishing Legitimacy

Multiple steps were taken to ensure that the community’s spaces and preferences were respected. First, representatives from partnering organizations assisted in identifying administrative leaders and community advocates for the target populations. In some instances, building trust required several connection attempts, attending various events to reach out and establish a trusting, genuine relationship. It was important to establish clear communication channels with leaders and advocates and identify their preferred methods of communication (e.g., phone calls, messages, emails, etc.). In engaging with community advisory members, phone calls emerged as the preferred method of communication. This preference was due to some individuals relying on their children to access emails or not regularly checking their personal email accounts. It was critical to learn about community norms, be humble, assume that we knew nothing about the communities, ask for clarifications when needed, and include justifications for our questions. For instance, our community healthcare partnering organizations informed us that the Rohingya language is exclusively spoken, which was new information for us. Consequently, during our advisory meeting, community members suggested creating a video for advertising purposes instead of using flyers for the Rohingya community. During the interview process, it was important to remain open to participants’ questions and share about ourselves as humans, and not only as academics. A key element was discussing how the research project benefits their communities to create a transparent message about what we brought as well as what we were asking for. We requested permission from community administrative leaders prior to approaching organizations, participating in events, or interacting with community members. We also prioritized giving leaders adequate time and autonomy to approve our study procedures, whether in their original form or adapted according to their guidance. Empowering communities with a leadership role through shared decision-making was an important part of this process: (1) partnering organizations were engaged early in the process to help formulate the research focus and define the characteristics of the study sample; (2) the advisory group members shaped the research questions and informed the advertisement process; (3) the research team worked around the community organizations’ schedules and preferences, and followed their guidance around the logistics that worked best for them. When in their community, it was important for the research team to continue to respect their norms and preferences and apologize for any misunderstandings. These actions were essential for maintaining a trust-based relationship with community partners. For instance, when the team was in a community’s mosque, the plan was for the leader to announce the study after the Friday sermon, then community members could approach us to learn more. However, people started leaving the main room and approaching our team before the leader’s announcement was over. We apologized to the leadership team for our inability to manage the timing of community interactions until the leader’s announcement was completed. When leaving the community, it was equally important to speak positively and respectfully of them, keeping their secrets, continuing to connect with them regularly, and bringing back the research results to share. The team engaged with a community health center that serves the refugee population to present preliminary findings from the parent study. This consultation helped the health center to gain insights into the challenges faced by community members and facilitated a collaborative brainstorming session to develop strategies for improving healthcare accessibility for this population.

An example of engaging with the Rohingya community. In connecting with the Rohingya leaders, researchers leveraged relationships through the Chief Executive Officers (CEOs) of the partnering healthcare community organizations. One person connected the team with a refugee case manager and the Rohingya mosque leader. The other CEO connected the team with a bilingual Rohingya interpreter who is the niece of the mosque leader. This interpreter recorded the video in the Rohingya language to promote the study within their community. The refugee case manager had integral roles in the advisory group discussions and in further connecting the team with the mosque leader. The research lead, A.A., contacted the mosque leader, introduced herself, explained the mission of the research, and outlined her role. This allowed A.A. to learn about the community, its norms, activities, and the mosque’s structure. This provided an opportunity to gauge their interest in the research and whether involving community members would be beneficial. The leader approved of the team to approach the community after the Friday prayer sermon, scheduled for a designated room within the mosque. The leader explained that this event is mainly attended by male individuals. Therefore, with respect to the community’s mosque structure and religious preferences, it worked well to involve the leader’s niece in the recruitment activity as a trusted community member and an interpreter.

An example of engaging with the Afghan community. The research team engaged with communities by participating in community events and volunteering in activities. For example, A.A. helped with women’s activities at a program tailored for Afghan refugees and expressed interest in continuing to help with these events. Community members were able to connect with the researcher and build a genuine human relationship that supported people’s interest in participating in this research, when previously the same people did not show interest in response to an earlier advertisement on the community’s WhatsApp chat group. To be in this space, A.A. used the same strategy described earlier in approaching communities.

#### 4.1.3. Leveraging Personal Connections for a Direct-to-Participant Recruitment Process

After the initial contacts, ongoing personal connections within the community were a valuable resource for recruitment. A.A. leveraged personal connections with a physician who previously supported the Michigan Muslim community and a pharmacy owner in Milwaukee to inform specific community members about the research and encourage them to participate if interested. Leveraging existing connections to establish a trustworthy relationship was one of the most successful recruitment strategies; five people were recruited through these two connections.

#### 4.1.4. Respecting Community’s Decisions

Levels of comfort with the research process varied across communities. For instance, people from the Somali community were hesitant to share their personal information and had concerns about privacy. To address this, the research team made multiple efforts to build trust, including repeated outreach and consultations with researchers experienced in working with Somali communities in Minnesota to gather culturally informed recommendations. Despite these efforts, the team had to step back and respect the community’s decisions about participation in research, in keeping with ethical principles of autonomy and community-driven engagement.

## 5. Discussion

This paper outlined methods for conducting qualitative research with a multilingual, heterogeneous sample of Muslim participants; examined recruitment strategies and the importance of cultural sensitivity; and reflected on considerations for sampling distinctly different Muslim populations simultaneously. Table 1 provides a summary of recommendations that can guide future studies in designing culturally responsive and sustainable partnerships with Muslim communities.

Maintaining these trust-built relationships over time may involve periodic check-ins with community leaders and partnering organizations, sharing study updates and findings in accessible formats, and sustaining a presence in community spaces in ways that reflect each community’s preferences. Identifying opportunities for mutually beneficial future collaborations and supporting community-driven priorities can further reinforce trust and strengthen the long-term connections established through this work.

Seclusion or Khalwah refers to a situation where a man and a woman, who are not closely related (non-mahram), are alone together in a private space [24]. This is an important consideration when conducting research with Muslim communities. To ensure cultural alignment and maintain participant comfort, the research team incorporated methodological safeguards to avoid situations that could be perceived as seclusion. Across sites, this involved coordinating with community leaders in advance to secure appropriate meeting spaces, arranging for the presence of additional individuals when gender-discordant interactions occurred, or keeping doors partially open during interviews [25]. Consistent with previous research, it was important to ensure gender concordance between research participants and the interviewer [26]. Understanding the religious concept behind it supported researchers in appreciating these preferences by community members.

Interview dynamics differed substantially depending on whether interpretation was required. Interviews that were conducted with Arabic-speaking and English-speaking participants flowed in a smoother manner, took less time, and were less exhausting for the interviewer. However, the depth of interviews did not differ across the various interpreter modalities. There are multiple challenges to using interpretation services over having a bilingual researcher conducting interviews. Challenges include interpreting information accurately, the time and effort needed, and securing resources and funding for interpretation costs. Misinterpretation and incomplete or inaccurate translation are well-documented in the literature [27]. These challenges are also frequently observed in clinical practice, where limited English proficiency has been shown to affect access to care [10]. Researchers are recommended to check for certifications and other indicators of qualification when using an interpreter from the community [28], in addition to staying alert for cues that may indicate inaccurate interpretations. By-phone interpretation offers a cheaper hourly rate; however, concerns about qualifications and providing accurate interpretation still apply. Technical challenges, such as call disruptions and unexpected disconnections, are expected to arise. The structure of the phone interpretation services, based on interpreter availability, complicates the process of securing a specific interpreter or reconnecting with a previously engaged one after a call drop. Call drops required the interviewer to reconnect, wait to be reassigned to a new interpreter, and then provide a brief orientation about the interpretation goals and expectations. These disruptions occurred because the phone-based interpretation system used for the study does not support extended interpretation sessions. All phone-interpreted interviews experienced at least one call drop; however, each interview was completed in full despite this technical challenge.

Overall, involving a language-concordant researcher appeared to facilitate the interview process. This aligns with evidence showing that language-concordant providers and in-person professional interpreters help foster greater trust in patient–provider communication [29]. Research in diabetes care further demonstrates that language-concordant interactions are associated with improved health outcomes [30,31]. Working with an in-person interpreter, especially from the community, can provide additional benefits such as leveraging trust-based connections to facilitate recruitment and understanding the culture. By-phone interpretation offers an affordable option; however, researchers need to brief the interpreter about the study and expectations, understand that interpreters are not able to see visual cues, and that technical challenges are anticipated [32].

Our efforts to engage the Somali community parallel earlier research, noting comparable challenges in recruitment and participation [26,33]. Researchers contacted community members multiple times, who expressed the need to include other community members to feel more comfortable participating in the study [33]. Researchers are encouraged to establish trust-based partnerships with community representatives, engage consistently throughout the project, and collaborate with institutions located in areas with larger Somali populations, such as Minnesota. Such approaches require adequate time and funding to support culturally grounded and community-aligned research practices.

### Limitations

This study has several limitations that should be considered. First, although the goal was to recruit 30 participants, the final sample included 22 individuals, which may limit the breadth of perspectives captured. Second, the research team was unable to recruit participants from the Somali community, highlighting the need for extended, trust-based engagement with some populations. Third, despite employing multiple interpretation approaches and briefing interpreters prior to interviews, interpreter-related bias remains a possible limitation, as variations in interpretation style or accuracy may have influenced communication and data quality. Furthermore, although most interviews were interpreted by professional service providers, no formal quality-control measures were implemented. Additional funding and time would likely be required to incorporate such procedures. Fourth, although the primary research partnership was based in Milwaukee, Wisconsin, and a substantial portion of engagement occurred there, researchers successfully conducted interviews with participants living in other states (including Virginia, Texas, Illinois, and Michigan), which improves geographic diversity but still does not allow for claims of national representativeness. Finally, researcher positionality, particularly cultural, linguistic, and religious alignment with some communities, may have shaped participant engagement and aspects of data interpretation. However, positionality and potential biases are addressed in greater detail in a separate study, which outlines the strategies the team used to mitigate these influences to the greatest extent possible [11].

## 6. Conclusions

This research highlighted the feasibility and importance of community-engaged research to help researchers collaborate with diverse communities in a single study. Key methodological lessons include the need to adapt recruitment and data-collection approaches, work closely with community leaders and networks, and integrate cultural and gender-related norms into research procedures. These insights highlight important considerations for future community-engaged research aiming to ethically and effectively partner with diverse and hardly reached populations.

## Figures and Tables

**Table 1:** Recommendations for designing culturally responsive and sustainable partnerships with Muslim communities.

Recommendation	Description
Engage early and maintain consistent communications	Initiate contact well before recruitment and sustain communication to build familiarity, credibility, and trust.
Prioritize cultural and religious sensitivity	Adapt interactions to community norms, including gender preferences and principles of seclusion.
Recruit language-concordant researchers and appropriate interpretation methods	Employ language-concordant researchers when possible and brief interpreters thoroughly to support clear communication.
Remain flexible and adapt methods to each community	Adjust recruitment, scheduling, and communication strategies based on each community’s preferences.
Build trust-based relationships and respect decisions not to participate	Approach collaboration with humility and be prepared to step back when communities decline participation.
Collaborate with local institutions and embedded organizations	Partner with institutions located in areas where specific Muslim groups cluster to strengthen access and cultural alignment.
Secure adequate time and funding	Allocate sufficient resources for trust-building, multilingual engagement, and collaboration.

## Data Availability

The original data related to this study are presented in a separate article at https://doi.org/10.3390/diabetology6100104.

## Abbreviations

The following abbreviations are used in this manuscript:

CBPR	Community-Based Participatory Research
MENA	Middle Eastern and North African
LEP	Limited English Proficiency
CEOs	Chief Executive Officers
